# Dietary Quality and 6-Year Anthropometric Changes in a Sample of French Middle-Aged Overweight and Obese Adults

**DOI:** 10.1371/journal.pone.0087083

**Published:** 2014-02-06

**Authors:** Karen E. Assmann, Camille Lassale, Pilar Galan, Serge Hercberg, Emmanuelle Kesse-Guyot

**Affiliations:** 1 Université Paris 13 Sorbonne Paris Cité, UREN (Nutritional Epidemiology Research Unit), Inserm (U557), Inra (U1125), Cnam, Bobigny, France; 2 Département de Santé Publique, Hôpital Avicenne, Bobigny, France; University of Catanzaro Magna Graecia, Italy

## Abstract

**Background:**

Understanding the role of dietary quality in the progression of adiposity in populations already affected by overweight or obesity is crucial for the guidance of secondary prevention strategies.

**Objective:**

To examine the association of diet quality, as reflected by the French Nutrition and Health Programme (Programme National Nutrition Santé, PNNS) – Guideline Score (GS), with 6-year-changes in weight and waist circumference.

**Design and Methods:**

Subjects were 1029 male and 450 female participants of the SUplémentation en VItamines et Minéraux AntioXydants (SU.VI.MAX) cohort (1994–2002) with anthropometric variables at baseline and follow-up and available data for estimating diet quality at baseline. We employed analysis of variance and covariance models to investigate anthropometric changes (% of the initial value) by categories of the PNNS-GS, which contains both dietary components and a physical activity component, and of a modified score (mPNNS-GS) containing dietary components only.

**Results:**

In men, a low (<6 points) PNNS-GS was associated with greater 6-year weight gain (adjusted mean: 3.63% [95% confidence interval: 2.87%; 4.39%]) as compared to a high (≥9 points) PNNS-GS (2.10% [1.39%; 2.81%]); p = 0.01. Results for the mPNNS-GS were very similar. In women, no associations between diet scores and weight change were observed. No significant relation between dietary quality and change in waist circumference was present among either men or women.

**Conclusions:**

These results support a beneficial role of high dietary quality – as characterized by good adherence to official French nutritional guidelines – in secondary obesity prevention, among men.

## Introduction

A rapidly growing part of the population worldwide is affected by overweight or obesity [1], [2]. Primary and secondary obesity prevention (i.e. preventing an unfavourable progression of already existing overweight) are both crucial to prevent well-known comorbidities such as diabetes, cardiovascular disease and certain types of cancer [1]–[4]. The promotion of recommendations on diet and physical activity – two major modifiable factors related to body weight status – is a core element of public prevention strategies adopted by many countries [5], [6]. In France, the National Nutrition and Health Program (Programme National Nutrition Santé, PNNS) was initiated in 2001 to elaborate official dietary and physical activity recommendations, to disseminate them to the general public, and finally to coordinate diverse measures aimed at facilitating adherence to these guidelines in everyday life [7].

In epidemiological research, the development of indices to estimate dietary quality [8] has notably extended the possibilities to examine nutritional recommendations with respect to their potential impact on health determinants and disease outcomes. So-called “a priori methods” rely on the construction of scores reflecting adherence to recommendations or other dietary concepts, such as the Mediterranean diet [9], [10]. Prospective studies investigating the relation between diet scores and anthropometric indicators tend to show that higher dietary quality is associated with favourable outcomes, despite some inconsistency [11]–[18]. In particular, two studies have indicated a beneficial role of adherence to French recommendations on diet and physical activity concerning long-term changes in body weight and central adiposity [19], [20].

So far, no prospective epidemiological study has specifically targeted a sample of overweight or obese subjects in order to gain further insight into the role of a priori- defined diet quality in secondary obesity prevention. Yet, a closer investigation of this population, especially at risk of experiencing health problems in the case of further weight gain, is highly warranted. Thus, the aim of this study was to examine the association of diet quality and physical activity levels, as reflected by a score measuring adherence to French recommendations (the PNNS – Guideline Score, PNNS-GS), with 6-year changes in weight and waist circumference (WC), in French overweight and obese adults.

## Methods and Procedures

### Study population

Subjects were overweight and obese participants of the Supplementation en VItamines et Minéraux AntioXydants (SU.VI.MAX) study, whose initial objective was to assess the effect of a daily supplementation with antioxidant vitamins and minerals at nutritional doses on the incidence of cardiovascular diseases, cancers and overall mortality, using a double-blind, placebo-controlled, randomized design with a follow-up of eight years (1994–2002). Details on this study have been reported elsewhere [21]. Briefly, after a national recruitment campaign with a call for volunteers living in France (women aged 35–60 years or men aged 45–60 years), 21 481 subjects were willing to participate and returned a completed baseline questionnaire and written informed consent. Of these, 13 017 met the study's eligibility criteria (lack of disease likely to hinder active participation or threatened 5-year survival; acceptance of the possibility to be given a placebo and acceptance of the constraints of participation; lack of previous regular supplementation with any of the vitamins or minerals in the supplement provided; absence of extreme beliefs or behaviour regarding diet) and were present at the inclusion visit. After exclusion of 6 subjects outside of the desired age range and of 270 subjects who had immediately withdrawn consent, 12 741 subjects (5028 men and 7713 women) were included in the final study sample.

The SU.VI.MAX study was approved by the Ethics Committee for Studies with Human Subjects at the Paris- Cochin Hospital (CCPPRB °706) and the ‘Commission Nationale de l'Informatique et des Libertés’ (CNIL °334641). Starting from inclusion, participants were invited to undergo either a biochemical or clinical examination on a yearly basis.

### Dietary assessment

One 24-h dietary record was requested every 2 months, starting from baseline (1994). If the participants wished so, they also had the possibility to enter food records more frequently. The 24-h records consisted of self-reporting any food and beverage consumption occurring over a 24-h period, using the Minitel Telematic Network, small terminals (similar to personal computers) widely used in France at the beginning of the 1990s. In the case of incoherent reported caloric intake, dietitians inquired complementary information via telephone. Moreover, dietitians gave assistance to volunteers via telephone when they encountered problems with data entry. The days of the 24-h records were determined in advance so that weekly and seasonal variation could be taken into account. To facilitate the evaluation of food portion sizes, participants were provided with an instruction manual that included validated photographs of more than 250 typical French foods shown in three different portion sizes [22]. Intermediate portion sizes could also be chosen, leading to a total of seven possible portion sizes. Nutrient intakes were calculated using the SU.VI.MAX food composition table, which included more than 900 different foods [23].

For this prospective analysis, we averaged – for each subject – data on nutrient and food group consumption from all eligible 24-h records during the first 26 months of the study (1994–1996) as a measure for baseline diet. While single 24-h records may not accurately reflect usual dietary behaviour, averaged nutritional intakes from repeated 24-h records can be regarded as a proxy for habitual dietary intakes [24]. Information on the consumption of alcohol and seafood was not obtained by 24-h records but by questionnaires, as certain particularities have to be considered (amongst other things, these food groups tend to be consumed less frequently than others). Alcohol intake (grams of ethanol per day) was estimated using a short, validated, semi-quantitative dietary questionnaire. In the validation study, there was a high concordance between the self-administered questionnaire and a dietetic interview using the dietary history method (correlation coefficients for alcohol: r = 0.80 in men and r = 0.75 in women) [25]. Specific information on weekly consumption of seafood was collected by a self-administrated questionnaire at baseline.

### Physical activity

Physical activity was assessed in 1998, using a French validated, self-administered version of the Modifiable Activity Questionnaire (MAQ). In the French validation study, a high level of concordance was shown for self-administration vs. interview (r = 0.90 for the leisure activity subscore that we considered in our analyses) [26]. In validation studies of the original questionnaire, the leisure activity subscore had a correlation of r = 0.56 with total energy expenditure (measured by double-labelled water) divided by basic metabolic rate [27]; and of r = 0.62 with counts from an electronic single-plane accelerometer [28].

In the SU.VI.MAX study, type, frequency, and duration of leisure time activities performed at least 10 times during the past 12 months (with a minimal duration of 10 minutes per session) were collected. Using published compendiums [29], [30], we assigned metabolic equivalent task-hours to each activity reported and computed average metabolic equivalent task-hours per week of physical activity.

### Anthropometric data

Anthropometric measurements were performed by trained technicians, at the first (1995–1996) and last (2001–2002) clinical examinations during follow-up. Weight was measured with an electronic scale (Seca, Hamburg, Germany), with subjects wearing indoor clothing and no shoes. Height was measured under the same conditions with a wall-mounted stadiometer. Waist circumference was measured as the circumference midway between the lower ribs and iliac crests, in a standing position and with an inelastic tape.

### Covariates

Data on gender, date of birth, tobacco use status and educational level were collected at study inclusion, using a self-administrated questionnaire. Information on menopausal status was obtained by questionnaires.

### Selection of participants for the present analyses

**Figure 1:**
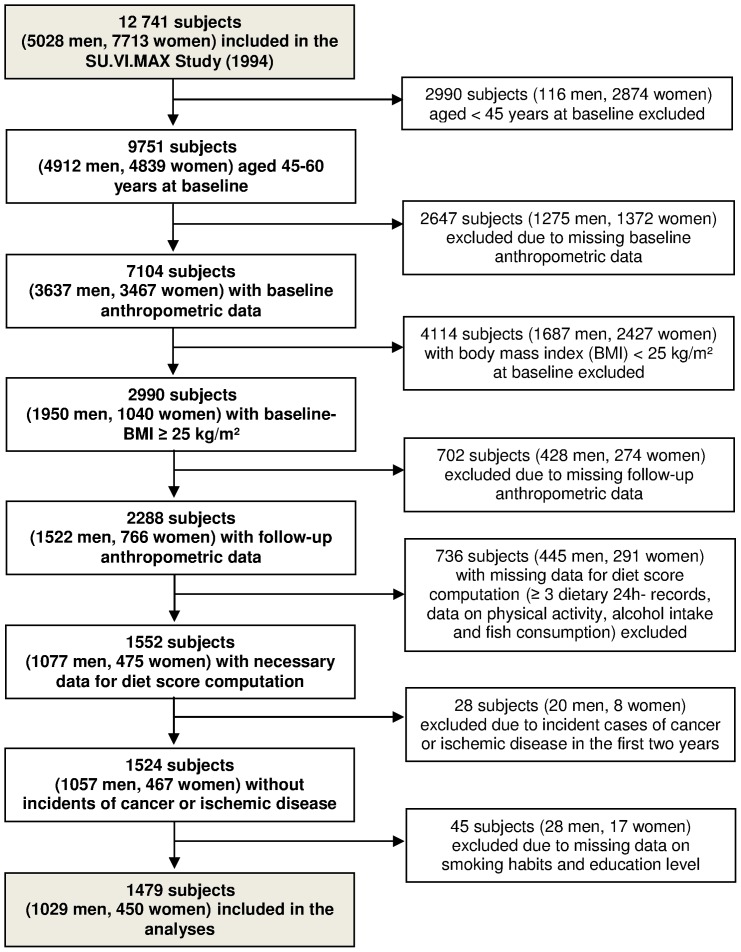
Selection of participants of the SU.VI.MAX study, France, 1994–2002, for the present analyses.

**Figure 1** shows the selection of participants for the present analyses. We chose to only include subjects aged 45–60 years at baseline in order to obtain a more homogenous sample; n = 9751 subjects of the original SU.VI.MAX study sample met this criterion. Measured body mass index (BMI) at baseline was available for 7104 of these subjects, and n = 2990 individuals were overweight or obese (measured BMI ≥25) at baseline. Of these, we included all subjects with at least three 24-h dietary records provided during the first two years of follow-up (1994–1996), who had no missing dietary, anthropometric or covariate data. Subjects with incident cases of cancer or ischemic disease during the first two years of follow-up were excluded as such incidents are likely to provoke changes in dietary habits and weight status. This resulted in a final study sample of 1479 participants (1029 men and 450 women).

As is the general rule in the SU.VI.MAX cohort [31], dietary records that reported <100 or >6000 kcal per day were considered implausible and were thus excluded from analyses. Further, men reporting <800 kcal per day and women reporting <500 kcal per day across more than one third of their dietary records were also excluded. In the final study sample, there were, on average, 10 dietary records available for each participant (median and mode of the number of dietary records: 11 and 13, respectively; range: 3–20). The proportion of participants with only 3 records or with more than 14 records was very low (4.4% and 1.1%, respectively).

### Data computation and statistical analysis

#### PNNS-GS computation

PNNS-GS computation, including food grouping, serving sizes, scoring, cut-off values and penalties, has previously been described in detail [32]. Briefly, the score includes 13 components and has a range of 0–15 points. Eight components refer to French food serving recommendations, four concern nutrients and food groups whose consumption is to be limited, and one component covers adherence to physical activity recommendations.

Scoring and cutoff values are presented in **Table 1**. A penalty for overconsumption was assigned to individuals whose energy intakes were higher than estimated energy needs [32].

**Table 1:** Construction of the Programme National Nutrition Santé – Guideline Score (PNNS-GS).

		Recommendation^1^	Scoring criteria^2^	Score
**1. Fruits and vegetables**	At least 5/d		[0–3.5]	0
			[3.5–5]	0.5
			[5–7.5]	1
			≥7.5	2
**2. Bread, cereals, potatoes**	At each meal according to		[0–1]	0
**and legumes**	appetite		[1]–[3]	0.5
			[3]–[6]	1
			≥6	0.5
**3. Whole grain food**	Choose whole grains and		[0–1/3]	0
	whole-grain breads more		[1/3–2/3]	0.5
	often		≥2/3	1
**4. Milk and dairy**	3/d (≥55-years-old: 3 to		[0–1]	0
**products**	4/d)		[1–2.5]	0.5
			[2.5–3.5] (55-years-old: [2.5–4.5])	1
			>3.5 (55-years-old: >4.5)	0
**5. Meat, poultry seafood**	1 to 2/d		0	0
**and eggs**			[0–1]	0.5
			[1]–[2]	1
			>2	0.5
**6. Seafood**	At least 2/week		<2/week	0
			≥2/week	1
**7. Added fat**	Limit consumption		Lipids from added fat >16% EI^3^/d	0
			Lipids from added fat ≤16% EI/d	1
**8. Vegetable added fat**	Favour fat of vegetable origin		No use of vegetable oil or ratio vegetable oil/total added fats≤0.5	0
			No use of added fats or ratio vegetable oil/total added fats >0.5	1
**9. Sweetened foods**	Limit consumption		Added sugar from sweetened foods ≥17.5% EI/d	−0.5
			Added sugar from sweetened foods 17.5–12.5% EI/d	0
			Added sugar from sweetened foods <12.5% EI/d	1
**Beverages**				
**10. non-alcoholic**	Drink water as desired		<1l water and >250 ml soda/d	0
	Limit sweetened		≥1l water and >250 ml soda/d	0.5
	beverages: no more than 1		<1l water and ≤250 ml soda/d	0.75
	glass/d		≥1l water and ≤250 ml soda/d	1
**11. alcoholic**	Women advised to drink		Ethanol >20 g/d for women and >30g/d for men	0
	≤2 glasses of wine/d and		Ethanol ≤20 g/d for women and ≤30g/d for men	0.8
	≤3 glasses/d for men		Abstainers and irregular consumers (<once a week)	1
**12. Salt** 4	Limit consumption		>12g/d	−0.5
			[10]–[12] g/d	0
			[8]–[10] g/d	0.5
			[6]–[8] g/d	1
			≤6 g/d	1.5
**13.Physical activity**	At least the equivalent of		[0–30] min/d	0
	30 min/d of brisk walking		[30–60] min/d	1
			≥60 min/d	1.5

1 Recommendations of the Programme National Nutrition Santé.

2 Servings per day unless otherwise indicated.

3 EI: energy intake without alcohol.

4 Established according to French recommended dietary allowances.

Energy needs were estimated on the basis of basal metabolic rate (calculated according to Schofield [33]) and physical activity levels. If energy intake exceeded estimated energy needs by more than 5%, an identical fraction was deducted from the PNNS-GS. For example, an energy over-consumption of 10% would result in reducing a PNNS-GS of 7 points to 6.3 points. The rationale for this approach is to account for the fact that subjects with high energy consumption will more easily meet recommendations on food groups for which consuming a certain minimal amount or more is considered as ‘healthy’ [9]. High scores on the PNNS-GS should not reflect a high general food intake, but a balanced diet with adequate caloric intake. In order to give the reader the possibility to assess the impact of penalization on our results, we present analyses with an unpenalized PNNS-GS in a supplemental table (**Table S1**).

Compliance with physical activity recommendations was determined through the MAQ when available, considering that half an hour of moderate activity on five days a week was equivalent to 16.25 metabolic equivalent task-hours per week. When the MAQ information was missing (this was the case for 14% of participants), data were obtained from two items of another baseline questionnaire. The respective items inquired whether the subject had a regular physical activity – and if yes, whether they estimated this activity to be equivalent to ≥ one hour of walking per day). Subjects who stated having no regular physical activity were classed into the low physical activity group (<30 min/day of equivalent walking) and those who indicated having a regular physical activity – and more specifically one equivalent to walking ≥ one hour per day – were classed into the high physical activity group. Regression imputation was applied to class the remaining subjects into the low or medium physical activity groups.

#### Statistical analysis

Changes in weight and WC were computed as the difference between follow-up (2001–2002) and baseline (1995–1996) values, and stated as a percentage of the baseline value. In order to give information about the SU.VI.MAX participants who were not included in our analyses, we compared included and excluded participants at two stages of the selection process (shown in **Figure 1**), using the Kruskal-Wallis test and the Chi^2^ test. Firstly, we compared participants excluded due to missing measured baseline BMI (n = 2647) with participants not excluded at this stage (n = 7104). Secondly, we compared participants who were in the desired age range and overweight or obese at baseline, but excluded due to missing data and other criteria (n = 1511), with the participants finally included into our analysis (n = 1479).

Baseline characteristics of participants included in the analyses were presented according to categories of the PNNS-GS, and differences across categories were tested using the Kruskal-Wallis test and the Chi^2^ test. An exploration of the distribution of our main study outcome (6-year-weight-change) across quartiles and quintiles of the PNNS-GS indicated a non-linear relationship between the two variables. Accordingly, we decided to categorize the PNNS-GS and mPNNS-GS and to use analysis of variance and covariance (ANCOVA) models for our main analyses, providing adjusted means (least squares means) of our outcome variables by diet score categories. To account for multiple comparisons, the significance levels presented were corrected according to Dunnett [34].

The choice of appropriate diet score cut-offs was based on the following elements: Cut-offs should 1) represent a sufficiently broad range in PNNS-GS; 2) lead to groups with a sufficient number of participants; 3) fit the context in which the PNNS-GS was originally created and validated (mean ± standard deviation of the score in the validation sample [32]: 7.54±1.91 in men; 7.87±1.86 in women). Finally, the following cut-offs were chosen: <6 points (low); ≥6 and <9 points (medium); ≥9 points (high); with the low and high cut-offs approximately corresponding to the mean PNNS-GS within the original validation sample ± its standard deviation (SD): 7.5±1.5 points. Cut-offs for the mPNNS-GS were defined as <5.5 points; 5.5 to <8.5 points; and ≥8.5 points.

Because there was a significant interaction between the categorized PNNS-GS and gender (p = 0.01), all analyses were stratified by gender. We created three ANCOVA models: a crude model (A), a model adjusted for age and energy intake (B) and a fully-adjusted model (C), further adjusted for supplementation group, number of dietary records, initial height, education level, smoking, and (in women) menopausal status. For analyses concerning the mPNNS-GS, models B and C were also adjusted for physical activity. Concerning women, model C is presented for illustrative purposes only and has to be interpreted with caution given the high number of adjustment variables in relation to the small number of women in the “low score” group. Identical adjustments were used when modeling weight change and change in WC.

All statistical analyses were performed using SAS software (Release 9.1, SAS Institute Inc., Cary, NC, USA). All tests performed were two-sided and p<0.05 was considered significant.

## Results

### Comparison of included and excluded participants

The proportions of males and of non-smokers among subjects excluded due to missing measured baseline BMI (n = 2647) were slightly smaller as compared to subjects with baseline anthropometric data (n = 7104): 48.2% vs. 51.2% (p = 0.008) and 44.6% vs. 48.2% (p<.0001), respectively (data not shown). There were no significant differences concerning education level, baseline age and self-declared baseline BMI.

**Table 2** presents baseline characteristics of SU.VI.MAX participants included in our analyses (n = 1479), as compared to participants who were aged 45–60 years and overweight or obese at baseline, but excluded due to missing data and other criteria. The population of excluded participants had a slightly higher median (declared) baseline BMI and a lower proportion of males. Moreover, they were less frequent to have only primary education – but also less frequent to have university level (or equivalent) education. Concerning baseline age and tobacco use status, the two populations were comparable.

**Table 2:** Characteristics of participants included in the analyses (n = 1479), as compared to excluded participants (n = 1511)^1^.

	Included participants (n = 1479)		Excluded participants (n = 1511)		
	*n* 2	Median (Q1, Q3) or n (%)^3^	*n* 2	Median (Q1, Q3) or n (%)	P^4^
**Baseline age (years)**	*1479*	51.5 (48.2; 56.0)	*1511*	51.3 (48.0; 55.7)	0.5
**Declared baseline BMI (kg/m^2^)**	*1451*	26.4 (25.2; 28.0)	*1431*	26.7 (25.3; 28.7)	0.0002
**Male sex (%)**	*1479*	1029 (69.6)	*1511*	921 (61.0)	<.0001
**Education level (%)**	*1479*		*1473*		0.04
Primary education		407 (27.5)		374 (25.4)	
High school diploma		548 (37.1)		614 (41.7)	
University level or equivalent		524 (35.4)		485 (32.9)	
**Tobacco use status (%)**	*1479*		*1389*		0.1
Non-smoker		613 (41.4)		572 (41.2)	
Former smoker		705 (47.7)		626 (45.1)	
Smoker		161 (10.9)		191 (13.8)	

SU.VI.MAX Study, France, 1994–1996.

BMI: body mass index.

Q: quartile.

1 Parent population: participants of the SU.VI.MAX study with a baseline age of 45–60 years and a baseline body.

mass index of ≥25 kg/m^2^ (n = 2990).

2 Number of participants for which data were available.

3 Median (Q1, Q3) for continuous variables and n (%) for categorical variables.

4 Kruskal-Wallis-test for continuous variables and Chi^2^-test for categorical variables.

### Subject characteristics

Of 12,741 adults initially included in the SU.VI.MAX cohort, 1497 overweight and obese subjects (1029 men, 450 women) with a mean baseline age of 52.1 (SD: 4.6) and a mean baseline BMI of 27.8 (SD: 2.8) were finally included in the present analyses (**Figure 1**). The prevalence of obesity (BMI ≥30 kg/m^2^) in our sample was 17.1% at baseline (13.7% in men and 24.9% in women). About half of the women in our sample (51.8%) were post-menopausal at baseline.

In **Table 3**, we present baseline characteristics of participants according to gender and by categories of the PNNS-GS. Men and women in the highest category had the lowest energy intake, were oldest, least likely to report a low physical activity level and had the smallest baseline waist circumference. Moreover, women with the highest diet quality also had the smallest baseline BMI and were the least likely to be obese or abdominally obese. Non-smokers were more frequent in the highest PNNS-GS category only among men.

**Table 3:** Baseline characteristics of participants included into the analyses^1^.

	Men (n = 1029)				Women (n = 450)			
	Low PNNS-GS	Medium PNNS-GS	High PNNS-GS		Low PNNS-GS	Medium PNNS-GS	High PNNS-GS	
	(n = 195)	(n = 593)	(n = 241)	P^2^	(n = 40)	(n = 255)	(n = 155)	P^2^
**Diet**								
Daily energy consumption (kcal)	2795 (2414; 3150)	2443 (2095; 2797)	2242 (1947; 2557)	<.0001	2198 (1685; 2554)	1724 (1416; 2088)	1667 (1376; 1924)	<.0001
PNNS-GS (0–15 points)	5.05 (4.25; 5.47)	7.42 (6.75; 8.25)	9.80 (9.30; 10.50)	<.0001	5.18 (4.41; 5.75)	7.75 (7.00; 8.25)	9.80 (9.30; 10.50)	<.0001
mPNNGS-GS (0–13.5 points)	4.75 (4.05; 5.30)	6.80 (6.25; 7.50)	9.00 (8.05; 9.50)	<.0001	5.03 (4.32; 5.75)	7.30 (6.55; 8.00)	9.30 (8.75; 9.80)	<.0001
**Socioeconomic/-demographic data**								
Age (years)	50.9 (47.7; 54.8)	51.7 (48.6; 55.8)	53.4 (48.9; 57.1)	<.0001	48.8 (46.8; 54.5)	49.9 (47.2; 55.0)	52.3 (48.4; 56.5)	0.006
Education level (%)				0.1				0.1
*Primary education*	34.9	25.8	25.3		42.5	27.8	23.9	
*High school diploma*	32.3	38.1	36.9		37.5	36.9	39.4	
*University level or equivalent*	32.8	36.1	37.8		20.0	35.3	36.8	
**Lifestyle factors**								
Tobacco use status (%)				0.02				0.09
*Non-smoker*	26.2	31.0	36.9		62.5	65.1	63.2	
*Former smoker*	57.4	56.0	55.6		20.0	27.5	31.6	
*Smoker*	16.4	13.0	7.5		17.5	7.5	5.2	
Physical activity level (%)				<.0001				<.0001
*30 min walk per day* 3	88.7	51.3	19.1		90.0	72.9	46.5	
*30–60 min walk per day* 3	8.2	26.0	30.3		10.0	17.7	25.2	
*>60 min walk per day* 3	3.1	22.8	50.6		0.0	9.4	28.4	
**Anthropometric data**								
BMI (kg/m^2^)	26.9 (25.7; 29.0)	27.1 (25.9; 28.7)	26.8 (25.8; 28.4)	0.3	27.9 (26.6; 30.4)	27.6 (25.9; 30.9)	26.7 (25.8; 28.5)	0.006
BMI ≥30 kg/m^2^ (%)	16.4	13.5	12.0	0.4	30.0	30.2	14.8	0.002
WC (cm)^4^	96.0 (92.0; 101.0)	96.0 (91.0; 101.0)	95.0 (90.0; 99.0)	0.03	88.0 (82.5; 96.5)	87.0 (82.0; 94.0)	84.0 (80.0; 90.0)	0.0005
Abdominal adiposity (%)^4,5^	20.0	17.9	13.7	0.2	45.0	43.9	30.3	0.02

SU.VI.MAX Study, France, 1994–1996.

PNNS-GS: Programme National Nutrition Santé Guideline Score. Categories of the PNNS-GS: Low: <6 points; medium: ≥6 and <9 points; high: ≥9 points.

BMI: body mass index. WC: waist circumference.

1 Values are medians (quartile 1; quartile 3) or frequencies.

2 Kruskal-Wallis-test for continuous variables and Chi^2^-test for categorical variables.

3 On at least five days of the week.

4 Data on WC was only available for 878 men and 391 women.

5 Word Health Organization cut-off for a substantially increased risk of metabolic Complications (for Caucasians): >102 cm (men); >88 cm (women) [53].

### PNNS-GS in relation to anthropometric changes

**Table 4** shows 6-year changes in weight and waist circumference in relation to categories of the PNNS-GS by gender. Men with a high PNNS-GS had a significantly lower 6-year-weight-gain as compared to men with a low PNNS-GS. Adjusted means in the fully-adjusted analysis were 3.63% [95% confidence interval: 2.87%; 4.39%] vs. 2.10% [1.39%; 2.81%]; p = 0.01. No association was observed in women. Moreover, there were no significant relations between the PNNS-GS and change in WC among either men or women.

**Table 4:** Six-year anthropometric changes according to categories of the Programme National Nutrition Santé Guideline Score (PNNS-GS), n = 1479.

	Low PNNS-GS			Medium PNNS-GS			High PNNS-GS, ref.		
	LSmean^1^	95%-CI^2^	p^3^	LSmean^1^	95%-CI^2^	p^3^	LSmean^1^	95%-CI^2^	overall p^4^
**6-year-weight-change (%)**									
*Men (n = 1029)*		*(n = 195)*			*(n = 593)*			*(n = 241)*	
Model A^5^	3.35	2.63; 4.06	0.002	2.23	1.82; 2.64	0.4	1.76	1.11; 2.40	0.004
Model B^6^	3.45	2.72; 4.19	0.002	2.21	1.80; 2.62	0.4	1.74	1.08; 2.39	0.003
Model C^7^	3.63	2.87; 4.39	0.007	2.51	2.05; 2.97	0.5	2.10	1.39; 2.81	0.01
*Women (n = 450)*		*(n = 40)*			*(n = 255)*			*(n = 155)*	
Model A^5^	1.70	−0.55; 3.96	0.3	2.63	1.73; 3.52	0.3	3.58	2.44; 4.73	0.2
Model B^6^	2.20	−0.08; 4.47	0.5	2.52	1.64; 3.40	0.2	3.63	2.49; 4.77	0.3
Model C^7, 8^	2.37	0.01; 4.73	0.5	2.79	1.64; 3.93	0.3	3.79	2.41; 5.17	0.3
**6-year-change in WC (%)** 9									
*Men (n = 878)*		*(n = 157)*			*(n = 508)*			*(n = 213)*	
Model A^5^	1.51	0.59; 2.43	0.3	1.09	0.57; 1.60	0.7	0.72	−0.08; 1.51	0.4
Model B^6^	1.67	0.72; 2.62	0.2	1.07	0.56; 1.58	0.6	0.64	−0.18; 1.45	0.3
Model C^7^	1.97	1.00; 2.94	0.3	1.49	0.92; 2.06	0.6	1.10	0.23; 1.97	0.4
*Women (n = 391)*		*(n = 34)*			*(n = 221)*			*(n = 136)*	
Model A^5^	1.30	−1.48; 4.08	0.4	1.85	0.76; 2.94	0.3	3.14	1.75; 4.53	0.3
Model B^6^	2.12	−0.73; 4.97	0.8	1.83	0.75; 2.92	0.4	2.97	1.57; 4.37	0.5
Model C^7, 8^	2.31	−0.68; 5.29	0.8	1.99	0.55; 3.43	0.4	3.15	1.44; 4.86	0.5

SU.VI.MAX study, France, 1994–2002.

Categories of the PNNS-GS: Low: <6 points; medium: ≥6 and <9 points; high: ≥9 points.

1 Least-squares mean.

2 95%- confidence interval (corrected according to Dunnett).

3 T-test with Dunnett correction.

4 Overall F-test (analysis of variance and covariance model).

5 Unadjusted.

6 Adjusted for age, energy intake.

7 Model B + adjustment for supplementation group, number of dietary records, initial height, education level, smoking, menopausal status (women).

8 Presented for illustrative purposes, but to be interpreted with caution (high number of adjustment variables/small number of women with a low PNNS-GS).

9 WC: waist circumference. Measures of WC at baseline and follow-up were only available for 878 male and 391 female participants.

### mPNNS-GS in relation to anthropometric changes

Associations between 6-year anthropometric changes and the modified PNNS-GS are presented in **Table 5**. Again, high dietary quality (mPNNS-GS ≥8.5 points) was related to lower gains in weight as compared to low dietary quality (mPNNS-GS <5 points) among men. The results obtained were very similar to those for the PNNS-GS, with adjusted means being 3.50% [2.72%; 4.28%] vs. 2.03% [1.24%; 2.82%]; p = 0.03. In women, mPNNS-GS and weight change were not related. Moreover, there was no significant association between the mPNNS-GS and changes in WC among either sex.

**Table 5:** Six-year anthropometric changes according to categories of the modified Programme National Nutrition Santé Guideline Score (mPNNS-GS), n = 1479.

	Low mPNNS-GS			Medium mPNNS-GS			High mPNNS-GS, ref.		
	LSmean^1^	95%-CI^2^	p^3^	LSmean^1^	95%-CI^2^	p^3^	LSmean^1^	95%-CI^2^	overall p^4^
**6-year-weight-change (%)**									
*Men (n = 1029)*		*(n = 200)*			*(n = 636)*			*(n = 193)*	
Model A^5^	3.04	2.33; 3.75	0.02	2.29	1.89; 2.69	0.3	1.75	1.02; 2.47	0.04
Model B^6^	3.27	2.52; 4.02	0.007	2.24	1.85; 2.64	0.3	1.67	0.93; 2.40	0.01
Model C^7^	3.50	2.72; 4.28	0.02	2.55	2.10; 3.00	0.3	2.03	1.24; 2.82	0.03
*Women (n = 450)*		*(n = 29)*			*(n = 263)*			*(n = 158)*	
Model A^5^	0.56	−2.08; 3.20	0.1	2.70	1.82; 3.57	0.4	3.60	2.46; 4.73	0.1
Model B^6^	0.59	−2.10; 3.29	0.1	2.52	1.63; 3.41	0.3	3.47	2.33; 4.61	0.1
Model C^7, 8^	0.46	−2.33; 3.25	0.1	2.79	1.63; 3.96	0.4	3.70	2.31; 5.08	0.1
**6-year-change in WC (%)** 9									
*Men (n = 878)*		*(n = 160)*			*(n = 542)*			*(n = 176)*	
Model A^5^	1.25	0.34; 2.16	0.5	1.17	0.67; 1.66	0.4	0.62	−0.25; 1.49	0.5
Model B^6^	1.49	0.53; 2.46	0.3	1.13	0.63; 1.63	0.4	0.51	−0.38; 1.41	0.3
Model C^7^	1.91	0.92; 2.90	0.3	1.54	0.98; 2.10	0.4	0.94	−0.02; 1.89	0.4
*Women (n = 391)*		*(n = 25)*			*(n = 225)*			*(n = 141)*	
Model A^5^	1.04	−2.20; 4.27	0.4	1.74	0.66; 2.82	0.2	3.29	1.92; 4.65	0.2
Model B^6^	1.93	−1.46; 5.33	0.8	1.75	0.65; 2.86	0.2	3.10	1.71; 4.49	0.3
Model C^7, 8^	2.01	−1.52; 5.53	0.7	1.93	0.47; 3.40	0.2	3.30	1.60; 5.00	0.3

SU.VI.MAX study, France, 1994–2002.

Categories of the mPNNS-GS: Low: <5.5 points; medium: ≥5.5 and <8.5 points; high: ≥8.5 points.

1 Least-squares mean.

2 95%- confidence interval (corrected according to Dunnett).

3 T-test with Dunnett correction.

4 Overall F-test (analysis of variance and covariance model).

5 Unadjusted.

6 Adjusted for age, energy intake, and physical activity (<30 min of walk per day vs. ≥30 min of walk per day).

7 Model B + adjustment for supplementation group, number of dietary records, initial height, education level, smoking, menopausal status (women).

8 Presented for illustrative purposes, but to be interpreted with caution (high number of adjustment variables/small number of women with a low mPNNS-GS).

9 WC: waist circumference. Measures of WC at baseline and follow-up were only available for 878 male and 391 female participants.

## Discussion

In this sample of French middle-aged overweight and obese adults, participants generally gained weight (on average 2.50%) over a 6-year period. However, men with a low dietary quality (low mPNNS-GS) gained more weight over time than those with a high dietary quality. The same was true for men with a low level of diet quality and physical activity combined (low PNNS-GS).

To the best of our knowledge, our study is the first to specifically investigate the relation of a priori- defined diet quality with anthropometric changes among overweight or obese subjects. However, two studies do provide information on the role of changes in empirically defined dietary patterns in the larger context of secondary obesity prevention. In the Swedish Mammography Cohort, the beneficial role of a “healthy pattern” was stronger among obese women (mean age: 54.2 years) than among normal-weight or overweight women [35]. Moreover, Japanese participants of a health promotion program (mean age ≈60 years) aimed at weight reduction achieved especially high losses in weight when changing their eating habits from a “sweets, meats, dairy products and alcohol”-pattern to a “plant foods and seafoods”-pattern and when maintaining a “plant foods and seafoods”-pattern [36].

The other available longitudinal studies investigating diet quality in relation to anthropometric outcomes considered either anthropometric changes in mixed samples of participants (normal weight, overweight, and obese subjects considered together) [11]–[16], [19], [20], or the risk of becoming overweight or obese within samples of normal-weight participants [11], [12], [17]–[20]. Overall, studies investigating the association of scores reflecting adherence to nutritional guidelines with anthropometric changes support a favorable role of high dietary quality, in line with our results concerning men (despite some inconsistency [12], [14], [16]). In particular, two previous investigations of the SU.VI.MAX study showed a favorable role of higher PNNS-GS scores in terms of changes in body weight over different periods of time (6 years [20] and 13 years [19], respectively). In an Australian cohort (age range: 25–75 years), higher adherence to national recommendations was related to lower weight gain among males [13], and in a US-study (mean age ≈52 years), better adherence to a subset of the Dietary Guidelines for Americans was inversely related to weight gain among males and females [15]. Besides, studies investigating empirically derived food patterns in relation to anthropometric changes (without excluding participants with a BMI ≥25) consistently identified different types of ‘healthy patterns’ to predict smaller gains in weight, BMI or waist circumference [35], [37]–[39]. The above-cited studies did not apply scores including a physical activity component, and correspond thus the most to our analyses concerning the mPNNS-GS. Of note, in our study, results for the mPNNS-GS and the PNNS-GS were very similar and physical activity was not significantly related to anthropometric changes (data not shown). This may be due to a potential bias caused by selective overreporting of physical activity by participants especially at risk of an unfavorable progression of overweight.

In our study, neither the mPNNS-GS nor the PNNS-GS were significantly related to anthropometric changes among women. In fact, women with a low diet quality even tended to gain less weight over time than women with a high diet quality. This unexpected relation was stronger for the mPNNS-GS than for the PNNS-GS as a higher physical activity tended to be associated with lower gains in weight among females (p for the crude association  = 0.2; data not shown). Supplementary analyses conducted to understand the lack of an association among females revealed that 8 out of 9 (89%) women with a low mPNNS-GS had reduced their energy intake by ≥350 kcal (baseline energy intake compared to intake at the end of follow-up, within a subsample of 197 women with ≥3 dietary records at the end of follow-up). Among women with a high baseline mPNNS-GS, such drastic changes were only present in 15% (12/79). Concerning women with a low vs. a high PNNS-GS, the respective proportions were 56% (10/18) and 18% (14/80). Accordingly, our unexpected findings in women could, at least in part, be explained by an active reduction of caloric intake, particularly in those in the lowest diet quality group. Considering the age range of women in our sample, hormonal changes linked to menopause could have acted as a confounding factor. However, baseline menopausal status was not significantly related to weight change in our sample (p in an ANOVA model adjusted for baseline age  = 0.5). Moreover, expressing weight change as an absolute value (follow-up weight – baseline weight) did not change the direction of our results for females, whether we adjusted for baseline weight or not (data not shown).

We conducted supplemental analyses in which we applied a more severe approach of excluding underreporters, using the Black/Goldberg method with physical activity level (PAL)- factors of 1.55, 1.70 and 1.85 (participants in the lowest, middle, and highest category of physical activity, respectively) [40]. The choice of PAL-factors was oriented by a joint FAO/WHO/UNO- report [41] and the indications given by Black [40]. In these analyses (with drastically reduced study samples of n = 301 women and n = 786 men), the unexpected results found for women were slightly less pronounced concerning weight change. Concerning change in WC, results were now in the ‘expected direction’ (women with a higher dietary quality gained slightly less WC over time than women with a lower dietary quality), but far from being statistically significant (n = 0.9). Our results concerning men were not substantially altered. In conclusion, underreporting by women especially at risk to gain weight may also partly explain our unexpected results for females.

Similar to our study, two previous studies have observed an association between dietary quality and anthropometric changes among men only [13], [19]. Reverse causality in relation to dieting (a behavior much more frequent in women than in men [42]) has been discussed as a probable explanation for this phenomenon [13]. In prior investigations of data from SU.VI.MAX participants of all BMI categories, a beneficial role of the PNNS-GS among women was found concerning 6-year-anthropometric changes [20], but not concerning 13-year-anthropometric changes [19]. This underlines that identifying determinants of weight change among women may be especially problematic in populations of overweight or obese subjects and in studies with very long follow-up periods (leaving time for changes in dietary behavior). Concerning our data, the results regarding women should be considered with much caution.

Contrary to previous research [12], [13], [32], we did not observe any association between diet quality and WC, neither among men, nor among women. One possible reason is that measurement error is generally higher for WC than for weight [43], especially among overweight and obese subjects [44]. In addition, mean gain in WC was only 1.44% over the six years of follow-up, making it potentially difficult to detect differences between groups. Furthermore, baseline and follow-up measurements of WC were only available for 1269 of the 1497 subjects in our study sample. Thus, statistical power was greater in analyses on weight than in analyses on WC.

Concerning the public health relevance of our results, it has to be noted that the observed effect size of our main finding is rather small: men with a low PNNS-GS gained 3.63% of their initial weight over six years while men with a low PNNS-GS only gained 2.10% (data not shown), corresponding to a mean difference of 1.53%. Yet, on the population level, even minor shifts in adiposity indicators could have an impact on chronic disease incidence [45], [46].

Several limitations of our study should be taken into consideration. First, the external validity of our results might be limited as the SU.VI.MAX cohort is a selected sample of compliant volunteers [21]. Notably, the study's participants may have had a lower the risk of weight gain as compared to the general population, and our sample may have been rather homogeneous in terms of diet quality.

Further selection bias could have occurred because of the high rates of exclusion of participants due to missing data (see **Figure 1**). While no sensitivity analysis could be conducted to assess potential bias due to exclusion of participants without measured baseline BMI, we did carry out supplemental analysis in order to account for possible bias due to the exclusion of subjects who had missing values on other variables (or incidents of chronic disease). Notably, these excluded subjects had a slightly higher self-declared BMI as compared to the subjects included into our analyses (see **Table 2**). We applied the “inverse probability weighting” method [47]–[49], consisting of two steps: The probability to be included in the present analysis was determined for each of the 2990 SU.VI.MAX participants who were overweight or obese and aged 45–60 years at baseline, using a logistic regression model with baseline characteristics as independent variables (missing values were dealt with by regression imputation). Then, data were reanalyzed using the inverse of these probabilities as weights. Weighted models yielded essentially the same results as our main models.

Secondly, most of the participants not included into our analysis because they were not overweight/obese at baseline were women (in line with the fact that less women than men have a BMI ≥25 in the French general population [50]). This drastically reduced the proportion of women in our study sample and resulted in limited statistical power concerning the analyses on female participants.

Thirdly, the construction of predefined diet scores is prone to subjectivity, notably concerning the selection of components, cutoff values, and their scoring system [9]. However, the PNNS-GS was created with the objective to closely reflect the entirety of French national guidelines, leaving less room for arbitrary choices, and has been shown to be a good predictor of nutritional status [32]. Fourthly, due to the observational design of our study, it is possible that our results are affected by residual confounding. In particular, we were not able to account for episodes of dieting or weight loss medication.

Important strengths of the present study include its prospective design and the high quality of anthropometric and dietary data: Anthropometric measurements were not self-reported but conducted in a standardized manner by trained technicians, strengthening their accuracy [51]. Dietary exposure was measured by repeated 24-h records, known to provide good estimates of intake at the individual level [52]. The availability of, on average, 10 records par participant permitted accounting for seasonal and weekly variability and thus assured a particularly high validity of dietary information. Moreover, the diet score applied in this study has the advantage that it accounts, unlike many other scores [9], for excess energy intake – a feature that is especially relevant when analyzing determinants of weight change (even if **Table S1** shows that penalization did not have a very large impact on our results).

### Conclusion

In this sample of overweight and obese French middle-aged adults, a low adequacy of the diet to French nutritional recommendations was related to a higher long-term weight gain among men. Thus, our findings suggest that, in men, diet quality has a role in the progression of overweight or obesity and confirm the importance of programs aimed at increasing adherence to national dietary recommendations, such as the PNNS.

## Supporting Information

Table S1 **Anthropometric changes according to categories of the unpenalized Programme National Nutrition Santé Guideline Score (PNNS-GS), n = 1479.** SU.VI.MAX study, France, 1994–2002. Categories of the (unpenalized) PNNS-GS: Low: <6 points; medium: ≥6 and <9 points; high: ≥9 points. ^1^ Least-squares mean. ^2^ 95%- confidence interval (corrected according to Dunnett). ^3^ T-test with Dunnett correction. ^4^ Overall F-test (analysis of variance and covariance model). ^5^ Unadjusted. ^6^ Adjusted for age, energy intake. ^7^ Model B + adjustment for supplementation group, number of dietary records, initial height, education level, smoking, menopausal status (women). ^8^ Presented for illustrative purposes, but to be interpreted with caution (high number of adjustment variables/small number of women with a low PNNS-GS). ^9^ WC: waist circumference. Measures of WC at baseline and follow-up were only available for 878 male and 391 female participants.
(DOC)
