# Supplementary material for: Dietary Quality and 6-Year Anthropometric Changes in a Sample of French Middle-Aged Overweight and Obese Adults
Source: PLoS One. 2014 Feb 6;9(2):e87083. doi: 10.1371/journal.pone.0087083 (PMC3916323; doi:10.1371/journal.pone.0087083)
Supplement: Table S1 — Anthropometric changes according to categories of the unpenalized Programme National Nutrition Santé Guideline Score (PNNS-GS), n = 1479. SU.VI.MAX study, France, 1994–2002. Categories of the (unpenalized) PNNS-GS: Low: <6 points; medium: ≥6 and <9 points; high: ≥9 points. 1 Least-squares mean. 2 95%- confidence interval (corrected according to Dunnett). 3 T-test with Dunnett correction. 4 Overall F-test (analysis of variance and covariance model). 5 Unadjusted. 6 Adjusted for age, energy intake. 7 Model B + adjustment for supplementation group, number of dietary records, initial height, education level, smoking, menopausal status (women). 8 Presented for illustrative purposes, but to be interpreted with caution (high number of adjustment variables/small number of women with a low PNNS-GS). 9 WC: waist circumference. Measures of WC at baseline and follow-up were only available for 878 male and 391 female participants. (DOC) [file pone.0087083.s001.doc]

**Table S1. Anthropometric changes according to categories of the unpenalized Programme National Nutrition Santé Guideline Score (PNNS-GS)**, n=1479

|  | **Low PNNS-GS** | | | **Medium PNNS-GS** | | | **High PNNS-GS, ref.** | |  |
| --- | --- | --- | --- | --- | --- | --- | --- | --- | --- |
|  | LSmean^1^ | 95%-CI^2^ | p^3^ | LSmean^1^ | 95%-CI^2^ | p^3^ | LSmean^1^ | 95%-CI^2^ | **overall p^4^** |
| **6-year-weight-change (%)** | | | | | | | | | |
| *Men (n= 1029)* |  | *(n= 170)* |  |  | *(n= 614)* |  |  | *(n= 245)* |  |
| Model A^5^ | 3.67 | 2.90; 4.43 | 0.0005 | 2.17 | 1.77; 2.58 | 0.5 | 1.80 | 1.17; 2.44 | 0.001 |
| Model B^6^ | 3.67 | 2.89; 4.45 | 0.001 | 2.17 | 1.77; 2.57 | 0.5 | 1.81 | 1.16; 2.46 | 0.001 |
| Model C^7^ | 3.85 | 3.05; 4.65 | 0.003 | 2.47 | 2.01; 2.92 | 0.6 | 2.16 | 1.46; 2.86 | 0.003 |
| *Women (n= 450)* |  | *(n= 29)* |  |  | *(n= 254)* |  |  | *(n= 167)* |  |
| Model A^5^ | 2.12 | -0.53; 4.77 | 0.6 | 2.66 | 1.77; 3.56 | 0.6 | 3.33 | 2.22; 4.43 | 0.6 |
| Model B^6^ | 2.15 | -0.46; 4.76 | 0.6 | 2.53 | 1.65; 3.41 | 0.3 | 3.52 | 2.43; 4.62 | 0.3 |
| Model C^7, 8^ | 2.35 | -0.32; 5.02 | 0.6 | 2.80 | 1.65; 3.95 | 0.4 | 3.66 | 2.33; 4.99 | 0.4 |
| **6-year-change in WC (%)^9^** | | | | | | | | | |
| *Men (n= 878)* |  | *(n= 134)* |  |  | *(n= 526)* |  |  | *(n= 217)* |  |
| Model A^5^ | 1.85 | 0.85; 2.84 | 0.2 | 1.00 | 0.49; 1.50 | 0.8 | 0.77 | -0.01; 1.56 | 0.2 |
| Model B^6^ | 1.92 | 0.90; 2.93 | 0.1 | 1.00 | 0.50; 1.51 | 0.8 | 0.71 | -0.09; 1.52 | 0.2 |
| Model C^7^ | 2.23 | 1.20; 3.26 | 0.2 | 1.42 | 0.85; 1.98 | 0.8 | 1.17 | 0.30; 2.03 | 0.3 |
| *Women (n= 391)* |  | *(n= 25)* |  |  | *(n= 219)* |  |  | *(n= 147)* |  |
| Model A^5^ | 1.44 | -1.80; 4.69 | 0.6 | 1.86 | 0.76; 2.96 | 0.4 | 2.97 | 1.64; 4.31 | 0.4 |
| Model B^6^ | 1.74 | -1.50; 4.99 | 0.7 | 1.82 | 0.73; 2.91 | 0.3 | 2.99 | 1.65; 4.32 | 0.4 |
| Model C^7, 8^ | 1.89 | -1.46; 5.23 | 0.7 | 2.01 | 0.56; 3.47 | 0.4 | 3.16 | 1.51; 4.81 | 0.4 |

SU.VI.MAX study, France, 1994-2002

Categories of the (unpenalized) PNNS-GS: Low: < 6 points; medium: ≥ 6 and < 9 points; high: ≥ 9 points.

^1^ Least-squares mean.

^2^ 95%- confidence interval (corrected according to Dunnett).

^3^ T-test with Dunnett correction.

^4^ Overall F-test (analysis of variance and covariance model).

^5^ Unadjusted.

^6^ Adjusted for age, energy intake.

^7^ Model B + adjustment for supplementation group, number of dietary records, initial height, education level, smoking, menopausal status (women).

^8^ Presented for illustrative purposes, but to be interpreted with caution (high number of adjustment variables/ small number of women with a low PNNS-GS).

^9^ WC: waist circumference. Measures of WC at baseline and follow-up were only available for 878 male and 391 female participants.
